# Risk of cancer after primary total hip replacement: The influence of bearings, cementation and the material of the stem: A retrospective cohort study of 8,343 patients with 9 years average follow-up from Valdoltra Orthopaedic Hospital, Slovenia

**DOI:** 10.1080/17453674.2018.1440456

**Published:** 2018-04-05

**Authors:** Vesna Levašič, Ingrid Milošev, Vesna Zadnik

**Affiliations:** 1 Valdoltra Orthopaedic Hospital, Ankaran; 2 University of Ljubljana, Faculty of Medicine, Ljubljana; 3 Jožef Stefan Institute, Ljubljana; 4 Institute of Oncology Ljubljana, Ljubljana, Slovenia

**Table 1.** Number of patients in sub-cohorts (bearing surface between head and cup, cementation, material of the stem) by age at operation

Group	Age group						
Subgroup	0–40	40–49	50–59	60–69	70–79	≥ 80	Total
All	131	591	1,631	3,059	2,664	267	8,343
Bearing							
MoM	12	43	141	124	18	0	338
MoP	39	233	763	2,128	2,481	265	5,909
CoC	69	274	535	407	38	0	1,323
CoP	11	41	192	400	127	2	773
Use of bone cement							
Non-cemented	119	580	1,596	2,910	1,713	48	6,966
Cemented	12	11	35	149	951	219	1,377
Stem material							
Ti-alloy	122	585	1,610	2,980	1,940	79	7,316
CoCr-alloy	9	5	21	67	586	162	850
Fe-alloy	0	1	0	12	138	26	177

